# Australian emergency department care for older adults diagnosed with low back pain of lumbar spine origin: a retrospective analysis of electronic medical record system data (2016–2019)

**DOI:** 10.1186/s12873-023-00789-8

**Published:** 2023-02-13

**Authors:** Katie de Luca, Andrew J McLachlan, Chris G Maher, Gustavo C Machado

**Affiliations:** 1 Discipline of Chiropractic, School of Health, Medical and Applied Sciences, CQUniversity, Brisbane, Australia; 2 Sydney Pharmacy School, University of Sydney, Sydney, New South Wales Australia; 3 Institute for Musculoskeletal Health, University of Sydney and Sydney Local Health District, Sydney, New South Wales Australia

**Keywords:** Low back pain, Lumbar, Emergency department, Hospital, Healthcare, Older people, Electronic medical records

## Abstract

**Background:**

In Australian emergency departments, 30% of all back pain presentations are for older adults. Relatively little is known about the care that this population receives during an emergency department stay, including admission to hospital. The aim of this study is to describe emergency department management of older adults diagnosed with a lumbar spine condition and to determine predictors of healthcare use in this population.

**Methods:**

A retrospective analysis of electronic medical record data of adults aged ≥ 65 years with a lumbar spine discharge diagnosis. Demographic, clinical care (date and time of presentation and discharge, length of stay in the emergency department, mode of arrival, triage category, re-presentations to the emergency department (within 48 h), discharge mode, the administration of pain-relieving medicines, lumbar imaging, and laboratory tests) and costs data were extracted from the electronic medical record system. Descriptive analyses and multilevel mixed-effects logistic regression models were performed.

**Results:**

Over the period January 2016 to December 2019 there were 4,093 presentations to emergency departments by older adults with a lumbar spine discharge diagnosis (82.0% were non-specific low back pain). Most were female (58.3%), 39.9% had some form of lumbar imaging, and 34.1% were admitted to hospital. The most administered pain medicines were opioid analgesics (67.1%), followed by paracetamol (63.9%) and NSAIDs (33.0%). Predictors of healthcare use and hospital inpatient admission were receiving a laboratory test and receiving any opioid. For the financial period 2019-20, the mean (SD) total cost of care per presentation was $5,629 ($11,982).

**Conclusion:**

In the emergency department, more than two thirds of older adults with a lumbar spine condition received opioid analgesics. They often received imaging and laboratory tests, had high costs and were admitted to hospital. Alternative pathways of care are needed to support older adults with low back pain, to receive guideline-concordant emergency department care and have good health outcomes.

**Supplementary Information:**

The online version contains supplementary material available at 10.1186/s12873-023-00789-8.

## Introduction

Low back pain (LBP) is the leading cause of disability, accounting for 64 million years lived with disability (YLDs) and the largest contributor to all-cause YLDs worldwide (7.4%). The prevalence of LBP increases over the lifespan with a peak rate observed at approximately 85 years of age [1]. In older adults, LBP is more severe than in younger age groups. For example, one in every four people aged > 80 years will report moderate to severe LBP and people aged > 80 years are three times more likely to have high intensity LBP (scores > 50, on a zero to 100 scale) than those aged 50–59 years [2, 3]. Being older is associated with non-recovery in adults with LBP, and, in a cohort of older adults presenting for a new primary care visit for back pain (that is, no healthcare visit for back pain within the prior 6 months), 77% had persistent back pain after 12 months [4]. Low back pain in older adults is associated with early retirement, [5] difficulties in caring for themselves at home, [6] increased disability, [7] and decreases their social well-being [6]. Despite the impact of LBP on older adults, [8] relatively little is known about the typical care provided to older people with LBP.

Back pain is ranked as the 5th most common reason for visiting an emergency department (ED) in Australia [9]. The pooled prevalence estimate for adults presenting to an emergency setting with low back pain is 4.39% (95% CI: 3.67–5.18) of all visits [10]. Re-presentations to a hospital emergency department (ED) for treatment of LBP is also common, with re-presentation rates within major cities 6%, inner regional EDs 8.8% and outer regional Eds 11.8% [11]. Management of LBP patients in the ED setting includes high rates of opioid analgesic use (69.6%), lumbar imaging (23.6%) and hospital admissions (17.6%) [12]. An Australian study of 6,393 presentations to the emergency department for LBP, noted that 30.9% were for older adults, aged ≥ 65 years [12]. In this study, non-specific LBP (82.3%) was the most common reason for presentation to the ED, followed by radicular LBP (12.4%) and serious spinal pathology (5.3%) – similar to the younger population. However, older adults with LBP were 2.8 times more likely to arrive by ambulance, 1.8 times more likely to receive lumbar imaging, and 3.0 times more likely to be admitted to the hospital, compared to younger adults [12].

While one third of emergency department presentations for LBP are by older adults, [12] how they are managed in the emergency department setting has been infrequently studied. Knowledge of the care received and factors associated with aspects of care (such as lumbar imaging, laboratory tests and pain management) may inform healthcare service planning and delivery for older adults with LBP. The aims of this study are to describe the emergency department management of older adults diagnosed with a lumbar spine condition; and to determine predictors of healthcare use (lumbar imaging, laboratory tests, analgesics and hospital inpatient admission) in this population.

## Methods

### Design

This is a retrospective analysis of electronic medical record data following the REporting of studies Conducted using Observational Routinely-collected Data (RECORD) guidelines (Supplementary Material 1) [13]. The Sydney Local Health District (Royal Prince Alfred Hospital zone) Ethics Committee granted approval for this study (protocol no. X17-0419).

### Study population

Patients aged ≥ 65 years who received a SNOMED CT-AU (EDRS) discharge diagnosis code related to a lumbar spine condition at any one of the three EDs within the Sydney Local Health District (Canterbury, Concord Repatriation General, and Royal Prince Alfred hospitals) from January 2016 to December 2019, were included. SNOMED codes were collapsed into three lumbar spine condition categories: LBP with non-specific cause (non-specific LBP), LBP with neurological signs and symptoms (radicular LBP), and LBP due to serious spinal pathology (serious LBP).

### Data sources

Re-identifiable data were extracted from the electronic medical record system using the secure, Sydney Local Health District Targeted Activity and Reporting System (STARS) to an Excel spreadsheet then to Stata/IC® (StataCorp, College Station, TX, USA) for analysis. STARS is a business intelligence program (Qlik Sense©, Lund, Sweden) integrated with the hospitals’ electronic medical records that provides reporting and analytical functions across multiple hospital datasets [14]. All patients seen in the Sydney Local Health District EDs have their episode of care recorded in the same electronic medical record system (Cerner PowerChart™, Kansas City, MO, USA), but imaging, laboratory tests, and medicines data are stored in separate databases. STARS links these hospital databases using the patient’s unique encounter identifier. The datasets used and/or analysed during the current study are available from the corresponding author on reasonable request.

### Data collection

Demographic, clinical care and costs data were extracted from STARS. All outcomes were reported under the categories of non-specific LBP, radicular LBP and serious LBP (due to serious spinal pathology). Demographic information comprised age, gender and socioeconomic status (derived from the postcode of the patient). We used the Australian Bureau of Statistic’s Socio-Economic Indexes for Areas 2016 [15] and reported as deciles, with the lowest decile designating areas with the highest socioeconomic disadvantage. Clinical care data comprised date and time of the emergency department presentation, discharge date and time, length of stay in the emergency department, mode of arrival (e.g., ambulance, public transport, walked in, private car), triage category, re-presentations to the emergency department (within 48 h), and discharge mode (subsequent inpatient admissions to hospital from the emergency department or discharged home). Data were extracted on the use of imaging and laboratory tests and treatments administered during their emergency department stay.

Triage categories, as assigned by the triage nurse based on the Australasian Triage Scale (ATS), [16] were also extracted. The ATS is a 5-point scale (1–5) where: 1 represents life-threatening condition requiring immediate response and resuscitation; 2 are emergency cases; 3 are urgent cases; 4 are semi-urgent cases; and 5 represents less urgent or clinical administrative problems requiring assessment and management to start within 2 h. Imaging was coded as plain radiographs, computed tomography (CT) and magnetic resonance imaging (MRI). Medications for pain management administered in the emergency department were classified according to the Anatomical, Therapeutic and Chemical (ATC) Classification System [17] categories (paracetamol, non-steroidal anti-inflammatory drugs (NSAIDs), muscle relaxants, corticosteroids, opioid analgesics, benzodiazepines, anticonvulsants, antidepressants, and antipsychotics) and combinations. Additionally opioid analgesics were stratified as single administrations of weak opioid analgesics (codeine or tramadol), strong opioid analgesics (buprenorphine, fentanyl, hydromorphone, morphine, oxycodone, pethidine or tapentadol) and combination prescriptions (any opioid analgesic with other single ATC categories).

We extracted patient-level costing data calculated for funding purposes by Sydney Local Health District according to the NSW Health Cost Accounting Guidelines [18]. This is presented as the mean total cost and costs for separate components of care, for the entire episode of care (i.e., both emergency department and inpatient care if admitted). Components of care includes clinical costs such medical, nursing, and allied health wages, as well as costs related to operating room care and critical care; imaging costs include plain radiographs, CT, and MRI, as well as radiography wage related costs; pathology costs include laboratory tests, such as blood or bodily fluid tests, as well as pathology wage related costs; medication costs include medicines administered, as well as pharmacy wage related costs; and other component of care costs include hospital overhead-costs, costs associated with facility maintenance, non-clinical and administration costs, surgical equipment and prostheses, ward supplies, emergency department supplies, average cost of all depreciation, staff leave, insurance cost and interest.

### Statistical analysis

Continuous variables are presented as mean (standard deviation (SD)) or median (inter-quartile range (IQR)) where appropriate. Categorical variables are presented as counts and percentages (%). Multilevel mixed-effects logistic regression models were used to account for the clustering effect of the hospital [19]. Firstly, patient characteristics (gender, age and socioeconomic status) and characteristics of the presentation (triage category, mode of arrival and duration of stay) were forced-entered as predictors in the multivariate models to explore associations with healthcare use (i.e., lumbar imaging, pain medications (single ATC groups and combination prescriptions of opioid analgesics with other single ATC categories), laboratory tests and discharge mode). The selection of these predictors was theory-driven, based on the available literature and on the expertise of the study team. Secondly, patient management (lumbar imaging, pain medication, laboratory tests) were forced-entered as predictors in multivariate models to explore associations with hospital inpatient admission. Data are reported as odds ratio (OR) and 95% confidence intervals (CI) and analyses were carried out in Stata version 14 (StataCorp, College Station, TX, USA).

## Results

### Sample characteristics

In the period January 2016 to December 2019 there were 4,093 presentations to the EDs by older adults who were diagnosed on emergency department discharge, with a lumbar spine condition. Counts and percentages (%) of all SNOMED discharge diagnosis codes can be found in Supplementary Material 2: Table S1. Characteristics of older adults diagnosed with low back pain of lumbar spine origin presentations are presented in Table 1. Most patients fell in the category non-specific LBP (3,358; 82.0%), followed by radicular LBP (523; 12.8%) and serious LBP (212; 5.2%). There were more female patients (2,384; 58.3%), and the proportion of women was highest in the radicular LBP category (323; 61.8%). The proportion of patients arriving by ambulance was highest in the non-specific LBP category (1,725; 51.4%), than serious pathology (94; 44.3%) and radicular LBP (181; 34.6%). Of all lumbar spine discharge diagnoses, 54.8% were triaged as semi-urgent or non-urgent (2,241), however 49.5% of serious LBP patients were triaged as urgent (105) and 3.8% as an emergency (8). Of all lumbar spine discharge diagnoses, 34.1% were subsequently admitted to hospital as an inpatient (1,144) and the serious LBP category had the highest proportion admitted to hospital (110; 51.9%). Patients in the non-specific LBP category admitted to hospital had a slightly longer inpatient length of stay (11.0; SD 13.6 days) than serious LBP (10.4; SD 11.8) and radicular LBP (9.4; SD 9.7 days).

**Table 1:** Characteristics of patients

Characteristics	Non-specific LBP (n = 3,358)	Radicular LBP (n = 523)	Serious LBP (n = 212)	All (n = 4,093)
Age, years – mean (SD)	77.3 (8.1)	76.3 (7.4)	77.8 (8.0)	77.2 (8.0)
Sex, female – no. (%)	1,960 (58.4)	323 (61.8)	101 (47.6)	2,384 (58.3)
Mode of arrival – no. (%)				
*Ambulance*	1,725 (51.4)	181 (34.6)	94 (44.3)	2,000 (48.9)
*Self-presented*	1,633 (48.6)	342 (65.4)	118 (55.7)	2,093 (51.1)
Triage category – no. (%)				
*Life-threatening*	0 (0.0)	0 (0.0)	0 (0.0)	0 (0.0)
*Emergency*	77 (2.3)	3 (0.6)	8 (3.8)	88 (2.1)
*Urgent*	1,491 (44.4)	168 (32.1)	105 (49.5)	1,764 (43.1)
*Semi-urgent*	1,758 (52.3)	344 (65.8)	96 (45.3)	2,198 (53.7)
*Non-urgent*	32 (1.0)	8 (1.5)	3 (1.4)	43 (1.1)
Emergency department LoS, hours – mean (SD)	5.0 (3.3)	4.2 (2.6)	5.9 (4.8)	5.0 (3.3)
Discharge mode – no. (%)				
*Inpatient admission* *Discharged home*	1,144 (34.1) 2,214 (65.9)	143 (27.3) 380 (72.7)	110 (51.9) 102 (48.1)	1,397 (34.1) 2,696 (65.9)
Inpatient LoS, days – mean (SD)	11.0 (13.6)	9.4 (9.7)	10.4 (11.8)	10.8 (13.2)
*Abbreviations: ED: Emergency Department; LoS: length of stay; LBP: low back pain; SD: standard deviation*				

Abbreviations: ED: Emergency Department; LoS: length of stay; LBP: low back pain; SD: standard deviation

### Lumbar spine imaging during the emergency department stay

Of the 4,093 presentations, 1,634 (39.9%) had some form of lumbar imaging. The serious LBP category had the highest proportion of any lumbar imaging (106; 50.0%), spinal radiograph (70; 33.0%), CT scan (60; 28.3%) and MRI (9; 4.3%) compared to the non-specific and radicular LBP categories (see Table 2).

**Table 2:** Lumbar imaging of patients

	Non-specific LBP (n = 3,358)	Radicular LBP (n = 523)	Serious LBP (n = 212)	All (n = 4,093)
Any lumbar imaging – no. (%)	1,396 (41.6)	132 (25.2)	106 (50.0)	1,634 (39.9)
Spinal radiograph – no. (%)	1,102 (32.8)	77 (14.7)	70 (33.0)	1,249 (30.5)
Advanced imaging– no. (%)				
*MRI*	49 (1.5)	11 (2.1)	9 (4.3)	69 (1.7)
*CT scan*	403 (12.0)	58 (11.1)	60 (28.3)	521 (12.7)

Abbreviations: MRI: magnetic resonance imaging; CT scan: computerised tomography scan; LBP: low back pain

### Pain medications during the emergency department stay

Electronic prescribing data were available for 2,901 presentations. Counts and percentages (%) of all individual medications administered during the patient’s emergency department stay can be found in Supplementary Material 3: Table S2. The most administered pain medicine was opioid analgesics (1,947; 67.1%), followed by paracetamol (1,854; 63.9%) and NSAIDs (956; 33.0%). Across all presentations, strong opioid analgesics were the most administered opioid – administered to 61.8% of the non-specific LBP category (1,465), 65.9% of the radicular LBP category (253) and 56.5% of the serious LBP category (83). Anticonvulsants were administered in 18.8% of the radicular LBP category (72), higher than that for the non-specific LBP category (232; 9.8%) and for the serious LBP category (6; 4.1%). The combination of an opioid *and* paracetamol was administered for 1,574 presentations (54.3%), while 676 presentations received opioids *and* NSAIDs (23.3%) and 278 received opioids *and* anticonvulsants (9.6%) (see Table 3).

**Table 3:** Analgesic medicines provided in the ED

	Non-specific LBP (n = 2,370)	Radicular LBP (n = 384)	Serious LBP (n = 147)	All (n = 2,901)
**Single pain medicines** – no. (%)				
Paracetamol	1,541 (65.0)	224 (58.3)	89 (60.5)	1,854 (63.9)
NSAIDs	799 (33.7)	128 (33.3)	29 (19.7)	956 (33.0)
*COX-2 selective inhibitors*	43 (1.8)	15 (3.9)	0 (0.0)	58 (2.0)
*Non-selective*	771 (32.5)	115 (30.0)	29 (19.7)	915 (31.5)
Muscle relaxants	15 (0.6)	3 (0.8)	0 (0.0)	18 (0.6)
Corticosteroids	98 (4.1)	14 (3.7)	7 (4.8)	119 (4.1)
Opioid analgesics	1,582 (66.8)	278 (72.4)	87 (59.2)	1,947 (67.1)
*Weak opioids*	227 (9.6)	41 (10.7)	7 (4.8)	275 (9.5)
*Strong opioids*	1,465 (61.8)	253 (65.9)	83 (56.5)	1,801 (62.1)
Anticonvulsants	232 (9.8)	72 (18.8)	6 (4.1)	310 (10.7)
Antidepressants	137 (5.8)	19 (5.0)	4 (2.7)	160 (5.5)
Antipsychotics	173 (7.3)	19 (5.0)	8 (5.4)	200 (6.9)
**Combined pain-relieving medicines** – no. (%)				
Opioids and paracetamol	1,287 (54.3)	211 (55.0)	76 (51.7)	1,574 (54.3)
Opioids and NSAIDs	552 (23.3)	100 (26.0)	24 (16.3)	676 (23.3)
Opioids and muscle relaxants	11 (0.5)	3 (0.8)	0 (0.0)	14 (0.5)
Opioids and corticosteroids	93 (3.9)	14 (3.7)	7 (4.8)	114 (3.9)
Opioids and anticonvulsants	208 (8.8)	66 (17.2)	4 (2.7)	278 (9.6)
Opioids and antidepressants	123 (5.2)	18 (4.7)	4 (2.7)	145 (5.0)
Opioids and antipsychotics	156 (6.6)	19 (5.0)	7 (4.8)	182 (6.3)
*Abbreviations: ATC: Anatomical Therapeutic Chemical Classification System; ED: Emergency Department; LBP: low back pain; NSAIDs: non-steroidal anti-inflammatory drugs*				

Abbreviations: ATC: Anatomical Therapeutic Chemical Classification System; ED: Emergency Department; LBP: low back pain; NSAIDs: non-steroidal anti-inflammatory drugs

### Laboratory tests during their emergency department stay

The top 10 laboratory tests ordered during the emergency department stay are shown in Table 4. In total, 43.7% or presentations were referred for any laboratory test (1,790). Full blood count (1,786; 43.6%) and urea electrolytes and creatinine (1,782; 43.5%) were the most common tests for all lumbar spine discharge diagnoses. Half of the serious LBP category were referred for urea electrolytes and creatinine (109; 51.4%) and full blood count (108; 51.0%) and the proportion of calcium, magnesium, phosphate tests were higher in the non-specific LBP category (970; 28.9%), than in the radicular LBP (108; 20.7%) and serious LBP categories (54; 25.5%).

**Table 4:** Laboratory testing

	Non-specific LBP (n = 3,358)	Radicular LBP (n = 523)	Serious LBP (n = 212)	All (n = 4,093)
Any laboratory test	1,507 (44.9)	174 (33.3)	109 (51.4)	1,790 (43.7)
*Full blood count*	1,504 (44.8)	174 (33.3)	108 (51.0)	1,786 (43.6)
*Urea electrolytes and creatinine*	1,500 (44.7)	173 (33.1)	109 (51.4)	1,782 (43.5)
*Liver function*	1,221 (36.4)	130 (24.9)	85 (40.1)	1,436 (35.1)
*Random glucose*	697 (20.8)	70 (13.4)	53 (25.0)	820 (20.0)
*C-reactive protein*	1,048 (31.2)	125 (23.9)	68 (32.1)	1,241 (30.3)
*Calcium, magnesium, phosphate*	970 (28.9)	108 (20.7)	54 (25.5)	1,132 (27.7)
*ED general*	347 (10.3)	30 (5.7)	27 (12.7)	404 (9.9)
*INR*	562 (16.7)	59 (11.3)	46 (21.7)	667 (16.3)
*AAPTT*	542 (16.1)	58 (11.1)	46 (21.7)	646 (15.8)
*Coagulation (INR, APTT)*	513 (15.3)	56 (10.7)	43 (20.3)	612 (15.0)

Abbreviations: APTT: activated partial thromboplastin time; ED: Emergency Department; INR: international normalised ratio; LBP: low back pain

### Costs of episode of low back pain care

From 824 presentations during the financial period 2019-20, the mean (SD) total cost of hospital care per presentation was $5,629 ($11,982). Mean (SD) total cost of care was highest for the non-specific LBP category ($5,844 ($12,754)), followed by the serious LBP ($4,620 ($5,325)) and then radicular LBP categories ($4,447 ($8,840)). Table 5 reports the total cost and separate cost components of LBP management for older adults in the emergency department and upon admission to hospital.

**Table 5:** Costs incurred by the hospital

Cost components	Non-specific LBP (n = 693)	Radicular LBP (n = 99)	Serious LBP (n = 32)	All (n = 824)
Clinical staff	3,048 (6,614)	2,293 (4,821)	2,635 (3,189)	2,942 (6,324)
Imaging	363 (820)	279 (613)	211 (296)	347 (784)
Pathology	215 (551)	165 (424)	208 (317)	208 (530)
Medication	124 (603)	62 (142)	44 (58)	114 (556)
Other*	2,092 (4,647)	1,646 (3,241)	1,519 (1,827)	2,016 (4,423)
**Total**	5,844 (12,574)	4,447 (8,840)	4,620 (5,325)	5,629 (11,982)

Abbreviations: LBP: low back pain; AUS: Australian dollar

*Costs are in AUD;* Clinical staff cost includes medical, nursing, allied health, critical care, and surgery

* includes: hospital overhead-costs, costs associated with facility maintenance, non-clinical and administration costs, surgical equipment and prostheses, ward supplies, emergency department supplies, average cost of all depreciation, staff leave, insurance cost and interest

Data are mean (SD) total cost per presentation and costs per component of care, for each low back pain category, in the 2019/2020 financial year

### Predictors of imaging, pathology tests and hospital inpatient admission

Table 6 reports the results of the multivariable regression models to predict factors associated with imaging, pathology tests and hospital inpatient admission. Older adults who received a laboratory test were more likely to be admitted as an inpatient to hospital (OR 48.06, 95% CI 35.66–64.77) and older adults who received any opioid were more likely to be admitted as an inpatient to hospital (OR 2.67, 95% CI 1.99–3.59). Older adults who had an emergency department triage of “urgent” were more likely to receive any imaging (OR 1.83, 95% CI 1.13–2.99) than those who had an emergency department triage of “emergency”. Older adults who had a radicular LBP presentation were more likely to receive an opioid (OR 2.65, 95% CI 1.71–4.12) than those with a non-specific LBP presentation. Older adults who arrived by ambulance were more likely to receive any laboratory test (OR 2.43, 95% CI 2.10–2.82).

**Table 6:** Predictors for imaging, laboratory tests, opioid use and inpatient admission

	Outcomes				
Predictors	Any imaging	Any opioid	Any laboratory test	Hospital admission	
Age	**1.05 (1.04–1.06)**	1.00 (0.99–1.01)	**1.05 (1.04–1.06)**	**1.03 (1.01–1.05)**	
Sex, Male	0.91 (0.80–1.04)	**0.83 (0.70–0.99)**	**0.83 (0.71–0.96)**	0.86 (0.67–1.10)	
Ambulance arrival	**1.29 (1.12–1.48)**	**1.76 (1.47–2.10)**	**2.43 (2.10–2.82)**	**1.55 (1.20–2.00)**	
SEIFA deciles 6–10	1.16 (0.99–1.36)	1.08 (0.87–1.33)	0.97 (0.81–1.15)	0.78 (0.57–1.07)	
ED triage category					
*Urgent*	**1.83 (1.13–2.99)**	0.75 (0.40–1.38)	**0.44 (0.26–0.74)**	1.03 (0.56–2.02)	
*Semi-Urgent*	1.58 (0.97– 2.58)	**0.53 (0.29–0.99)**	**0.23 (0.14–0.40)**	1.02 (0.51–2.04)	
*Non-Urgent*	**0.19 (0.04–0.85)**	**0.10 (0.02–0.41)**	**0.10 (0.03–0.32)**	1.49 (0.14–16.32)	
LBP diagnosis					
*Radicular LBP*	**0.41 (0.28–0.58)**	**2.65 (1.71–4.12)**	0.74 (0.50–1.09)	**0.28 (0.15–0.55)**	
*NSLBP*	**0.73 (0.54–0.99)**	**1.54 (1.05–2.25)**	0.79 (0.57–1.12)	**0.22 (0.12–0.39)**	
Received any opioid	**–**	–	–	**2.67 (1.99–3.59)**	
Received any image	**–**	–	–	1.14 (0.89–1.47)	
Received laboratory test				**48.06 (35.66–64.77)**	

Abbreviations: ED: Emergency Department; LBP: low back pain; CI: confidence interval; SEIFA: Socio-Economic Indexes for Areas

Results are reported as odds ratio (95% CI) for the multilevel mixed-effects logistic regression models where **bold** indicates statistically significant results

## Discussion

In older adults who presented to the emergency department with a lumbar spine condition, a non-specific LBP diagnosis was the most common discharge diagnosis and incurred the highest cost per presentation. Opioid analgesics were the most administered pain medication, with more than two thirds of patients being administered opioid analgesics. Additionally, more than half of all patients were administered combined opioids and paracetamol, nearly a quarter received opioids and NSAIDs, and 1 in 10 were given opioids and anticonvulsants. Older people arriving by ambulance were more likely to receive imaging, be administered opioids, be admitted to hospital and were nearly three times more likely to receive a laboratory test, than older adults who self-present to the emergency department.

Most of the patients received one or more aspects of care that is considered not concordant with evidence-based guidelines. For example, of the subset of older adults with non-specific LBP, 67% were prescribed opioids, 45% had a laboratory test, 42% received imaging and 34% were admitted to hospital. While our findings are limited by the lack of patient level data and we do not know the cause and characteristics of low back pain of lumbar spine origin, all four of these practices are discouraged in guidelines. Numerous clinical guidelines advise that people with LBP should initially receive non-pharmacological care, [20–22] with the greatest emphasis on advice to stay active, reassurance and education, [23] although specific guidelines for the management of LBP in the emergency department setting are lacking. Our findings demonstrated high rates of guideline-discordant care that have consequences for the patient (e.g., high use of opioids) and health system (e.g., high use of diagnostic testing and admission). An emphasis of future research should be placed on discovering strategies to successfully implement guideline concordant care for older adults in the emergency department setting.

Data analysed were collected for all LBP presentations to the emergency departments from hospitals’ electronic medical record systems between January 2016 to December 2019, with costs data extracted from the 2019/2020 financial year. While there was a decrease in the number of emergency department presentations of LBP during the COVID-19 pandemic, unnecessary aspects of emergency department care did not change, and therefore data collected during this time should not skew results [24]. A key strength of this study lies within the large, real world sample of older adults diagnosed at discharge with a lumbar spine condition in the emergency department (n = 4,093). A limitation of the study data set is that we did not have information on factors such as co-morbidities, physical activity, frailty, treatments used to manage LBP prior to emergency department arrival, medicines prescribed on discharge and patient-reported outcome measure data on pain intensity and disability, for example. Furthermore, data were collected from three metropolitan Sydney Local Health District EDs and may not be generalisable to other emergency departments.

The prevalence of serious LBP (due to spinal pathology) varies between settings. In primary care, rates of serious pathology are reportedly ~ 1%, while serious pathologies in patients with LBP who have been referred for MRI by in a private secondary care or public tertiary care are higher (3.2% and 14.8% respectively) [25]. Our report that 5% of older adults had LBP due to spinal pathology, is similar to another large Australian emergency department study (4.5%) [12] and concurs with previous research [26–28]. It has been recently reported that 16.2%, of older adults who were admitted to hospital, from the ED, with a provisional diagnosis of non-serious back pain were subsequently diagnosed with a specific spinal pathology, and a further 26.6% with serious pathology beyond lumbar spine [29].

The prescription of opioids for acute musculoskeletal pain in the emergency department has been associated with long-term opioid use, placing patients at risk for opioid dependence and overdose-related harms [30]. In this study, opioid analgesics were the most commonly administered pain medication, possibly due to a lack of alternatives for older adults who often have contraindications to simpler analgesics, [31] or that patient’s may have self-administered simple analgesics at home or prior to arrival at the emergency department. Also, 1 in 10 patients received opioids *and* anticonvulsants, which is not recommended and particularly dangerous in older people. Older people are at risk of adverse opioid effects because of a decline in renal and hepatic function, leading to reduced metabolism and clearance of medications [32], and opioids also contribute to an increased risk of falling, particularly in the first two weeks after prescription [33]. Alarmingly, rates of opioid overdose deaths continue to climb among adults 55 years and older [34]. It has been shown that in the emergency department there is no difference in the efficacy of non-opioid and opioid analgesic regimens for reducing acute musculoskeletal pain, but there were significantly more adverse events (nausea and vomiting) among patients treated with opioids [35]. While emergency department practice improvements are needed to appropriately prescribe opioids to older adults with a lumbar spine condition, future research should also focus on practical and safe alternatives to opioid administration to manage LBP in the ED.

While imaging is discouraged in guidelines, two in five older adults received some form of lumbar imaging, slightly higher than that reported for all patients in the ED [36]. Considering differences in the incidence of serious disease in older adults, higher rates of lumbar imaging may be expected [37]. For example, the prevalence of osteoporotic compression fracture as a cause of LBP is 6.5% in the emergency department setting, [38] and older adults are at a higher risk than younger patients, which may explain the high use of spinal radiographs.

Older adults with radicular LBP were more than two times more likely to receive opioids than those with non-specific LBP. Four clinical practice guidelines recommend a weak opioid as an option for radicular pain, with the Belgian Health Care Knowledge Centre advising against any opioid prescription for these conditions [39]. One in five older adults with radicular LBP were administered anticonvulsants, treatment which conflicts with evidence from a 2016 systematic review and meta-analysis that reported moderate to high-quality evidence that anticonvulsants were ineffective for radicular pain and that gabapentinoids had a higher risk for adverse events [40]. As the chief complaint of the ED visit in our cohort is back pain, it is likely that these medicines were administered to manage the condition, but we cannot rule out the possibility that they were used to manage a pre-existing condition. As older adults with radicular LBP were also less likely to receive imaging, management of radicular LBP is consistent with recommendations for care, even while recommendations themselves are inconsistent [39].

In the ED, 33% of older adults were administered NSAIDs and 23.3% were administered the combination of opioid analgesics and NSAIDs. Caution is warranted for older patients with back pain, especially those with renal impariment or heart faiulure and those already regularly taking NSAIDs or people treated with angiotensin-converting-enzyme inhibitors, diuretics, low-dose aspirin or anticoagulants [41]. This supports the careful use of NSAIDs in older people with low back pain at the lowest effective dose and for the shortest period needed with careful medical supervision [42].

Older adults are frequent users of the emergency department and their attendence is often influenced by lack of access to primary care, a lack of continuity of care, low income, comorbidity, frailty, polypharmacy and patient complexity [27, 43]. In this study, the length of emergency department stay was similar to that of Federico et al. [28]. The longest length of stay was for patients with serious LBP (six hours), which is higher than the average length of stay for LBP patients (four to five hours) [44]. This poses a significant burden on emergency department resources. While prescription of opioids for LBP in the emergency department is associated with an increased emergency department length of stay, [45] in this study 43% of older adults had a laboratory test and 40% had imaging while in the ED, which are also possible reasons for the longer duration of stay. There is also a propensity for older adults with acute LBP to be admitted to hospital, due to frailty, comorbidity and less carer support [46]. In this study, when older adults were admitted to hospital, the average length of stay was 11 days. While it has been shown a longer length of stay in hospital for older adults with LBP has been associated with a non-English speaking background, age 80 years and older, disc-related disease, vertebral fracture and sciatica, as well as the use physiotherapy, [46] care that is provided to patients in the hospital setting is unknown. As older adults with a lumbar spine condition are more likely to be admitted to hospital from the emergency department, a comprehensive description of reasons for admission, inpatient management and outcomes at discharge is warranted.

The high use of ambulance, as a mode of arrival, by older adults with LBP needs further investigation, to understand if social, psychological, medical and functional issues may justify this high use of ambulance resources. Care received during ambulance transport is unknown, and information on how this care could influence care for LBP in the emergency department is of interest. Alternate pathways of care that avoid emergency department presentations, hospital admissions and reduce length of stay in hospital, but provide good outcomes for older people with LBP are needed.

## Conclusion

In the emergency department, older adults with non-specific LBP often received imaging and laboratory tests, had high costs and were admitted to hospital. More than two thirds of older adults with a lumbar spine condition received opioid analgesics. Patient level data on the use of ambulance, reasons for admission, pre-existing conditions, and current use of pain medicines in this cohort is needed. Future research to develop alternative pathways of care to support older adults with LBP, to receive guideline-concordant emergency department care and have good health outcomes is essential.

## Electronic supplementary material

Below is the link to the electronic supplementary material.

Supplementary Material 1

Supplementary Material 2

Supplementary Material 3

## Data Availability

The datasets used and/or analysed during the current study are available from the corresponding author on reasonable request.

## Abbreviations

APTT: Activated partial thromboplastin time
ATC: Anatomical, Therapeutic and Chemical Classification System
ATS: Australasian Triage Scale
CI: Confidence interval
CT: Computed tomography
ED: Emergency department
INR: International normalised ratio
IQR: Inter-quartile range
LBP: Low back pain
LoS: Length of stay
MRI: Magnetic resonance imaging
NSAIDs: Non-steroidal anti-inflammatory drugs
OR: Odds ratio
RECORD: REporting of studies Conducted using Observational Routinely-collected Data
SD: Standard deviation
SEIFA: Socio-Economic Indexes for Areas
STARS: Sydney Local Health District Targeted Activity and Reporting System
YLDs: Years lived with disability.
