# Correction: Australian emergency department care for older adults diagnosed with low back pain of lumbar spine origin: a retrospective analysis of electronic medical record system data (2016–2019)

**DOI:** 10.1186/s12873-023-00834-6

**Published:** 2023-06-14

**Authors:** Katie de Luca, Andrew J McLachlan, Chris G Maher, Gustavo C Machado

**Affiliations:** 1 Discipline of Chiropractic, School of Health, Medical and Applied Sciences, CQUniversity, Brisbane, Australia; 2 Sydney Pharmacy School, University of Sydney, Sydney, NSW Australia; 3 Institute for Musculoskeletal Health, University of Sydney and Sydney Local Health District, Sydney, NSW Australia

**BMC Emergency Medicine (2023) 23:17**

10.1186/s12873-023-00789-8

The original article [1] contained a typographical error in Gustavo C Machado’s name which has since been amended.
