# Supplementary material for: Australian emergency department care for older adults diagnosed with low back pain of lumbar spine origin: a retrospective analysis of electronic medical record system data (2016–2019)
Source: BMC Emerg Med. 2023 Feb 13;23:17. doi: 10.1186/s12873-023-00789-8 (PMC9924838; doi:10.1186/s12873-023-00789-8)
Supplement: Supplementary file 2 — Supplementary Material 2 [file 12873_2023_789_MOESM2_ESM.docx]

**Supplementary Material**

Additional file 2: Supplementary Material 2.

Table S1: Counts and percentages (%) of all SNOMED discharge diagnosis codes (N=4,093).

| **SNOMED code description** | **Freq.** | **Percent** |
| --- | --- | --- |
| Backache (finding) | 1,918 | 46.86 |
| Low back pain (finding) | 1,274 | 31.13 |
| Sciatica (disorder) | 442 | 10.80 |
| Fracture of lumbar spine (disorder) | 107 | 2.61 |
| Abscess of back (disorder) | 53 | 1.29 |
| Sacral back pain (finding) | 39 | 0.95 |
| Acute low back pain (finding) | 26 | 0.64 |
| Lumbago with sciatica (finding) | 20 | 0.49 |
| Cauda equina syndrome (disorder) | 19 | 0.46 |
| Spinal stenosis of lumbar region (disorder) | 19 | 0.46 |
| Chronic back pain (finding) | 16 | 0.39 |
| Spasm of back muscles (finding) | 15 | 0.37 |
| Acute sciatica (disorder) | 12 | 0.29 |
| Chronic low back pain (finding) | 12 | 0.29 |
| Lumbar sprain (disorder) | 11 | 0.27 |
| Acute back pain with sciatica (finding) | 10 | 0.24 |
| Lower back injury (disorder) | 10 | 0.24 |
| Fracture of coccyx (disorder) | 9 | 0.22 |
| Lumbar radiculopathy (disorder) | 9 | 0.22 |
| Contusion of lower back (disorder) | 7 | 0.17 |
| Lumbar spondylosis (disorder) | 7 | 0.17 |
| Pain in the coccyx (finding) | 6 | 0.15 |
| Strain of back muscle (disorder) | 6 | 0.15 |
| Compression fracture of lumbar spine (disorder) | 5 | 0.12 |
| Chronic sciatica (disorder) | 4 | 0.10 |
| Crush fracture of lumbar vertebra (disorder) | 4 | 0.10 |
| Mechanical low back pain (finding) | 4 | 0.10 |
| Multiple fractures of lumbar spine and/or pelvis (disorder) | 4 | 0.10 |
| Blunt injury to back (disorder) | 3 | 0.07 |
| Contusion of back (disorder) | 3 | 0.07 |
| Degeneration of lumbar intervertebral disc (disorder) | 3 | 0.07 |
| Injury of lumbar nerve roots (disorder) | 3 | 0.07 |
| Lumbago-sciatica due to displacement of lumbar intervertebral disc (disorder) | 3 | 0.07 |
| Collapse of lumbar vertebra (disorder) | 2 | 0.05 |
| Sprain of ligament of lumbosacral joint (disorder) | 2 | 0.05 |
| Back pain complicating pregnancy (disorder) | 1 | 0.02 |
| Closed fracture lumbar vertebra (disorder) | 1 | 0.02 |
| Coccyx sprain (disorder) | 1 | 0.02 |
| Discitis (disorder) | 1 | 0.02 |
| Injury of sciatic nerve (disorder) | 1 | 0.02 |
| Stiff back (finding) | 1 | 0.02 |
| **Total** | **4,093** | **100.00** |
