# Supplementary material for: Australian emergency department care for older adults diagnosed with low back pain of lumbar spine origin: a retrospective analysis of electronic medical record system data (2016–2019)
Source: BMC Emerg Med. 2023 Feb 13;23:17. doi: 10.1186/s12873-023-00789-8 (PMC9924838; doi:10.1186/s12873-023-00789-8)
Supplement: Supplementary file 3 — Supplementary Material 3 [file 12873_2023_789_MOESM3_ESM.docx]

**Supplementary Material**

Additional file 3: Supplementary Material 3.

Table S2: Counts and percentages (%) of all individual medications administered during the patient’s emergency department stay

| **MEDICINES** | **N (%)** |
| --- | --- |
| **Paracetamol** | 1,854 (63.9) |
| **NSAIDs** |  |
| *Ibuprofen* | 497 (17.1) |
| *Aspirin* | 231 (8.0) |
| *Ketorolac* | 94 (3.2) |
| *Naproxen* | 76 (2.6) |
| *Indomethacin* | 51 (1.8) |
| *Diclofenac* | 36 (1.2) |
| *Parecoxib* | 31 (1.1) |
| *Celecoxib* | 27 (0.9) |
| *Diclofenac topical* | 8 (0.3) |
| *Ketoprofen* | 2 (0.1) |
| *Ibuprofen topical* | 1 (0.03) |
| **Muscle relaxants** |  |
| *Orphenadrine* | 15 (0.5) |
| *Baclofen* | 3 (0.1) |
| **Corticosteroids** |  |
| *Prednisolone* | 90 (3.1) |
| *Dexamethasone* | 21 (0.7) |
| *Hydrocortisone* | 8 (0.3) |
| *Prednisone* | 5 (0.2) |
| **Opioid analgesics** |  |
| *Oxycodone* | 1,641 (56.6) |
| *Morphine* | 268 (9.2) |
| *Paracetamol-codeine* | 202 (7.0) |
| *Oxycodone-naloxone* | 163 (5.6) |
| *Tapentadol* | 64 (2.2) |
| *Tramadol* | 50 (1.7) |
| *Fentanyl* | 45 (1.6) |
| *Buprenorphine* | 36 (1.2) |
| *Hydromorphone* | 31 (1.1) |
| *Codeine* | 22 (0.8) |
| *Methadone* | 6 (0.2) |
| *Dextropropoxyphene hydrochloride* | 1 (0.03) |
| **Anticonvulsants** |  |
| *Pregabalin* | 241 (8.3) |
| *Gabapentin* | 47 (1.6) |
| *Valproate* | 16 (0.6) |
| *Levetiracetam* | 10 (0.3) |
| *Carbamazepine* | 7 (0.2) |
| *Lamotrigine* | 1 (0.03) |
| *Phenytoin* | 1 (0.03) |
| *Zonisamide* | 1 (0.03) |
| **Antidepressants** |  |
| *Amitriptyline* | 41 (1.4) |
| *Mirtazapine* | 27 (0.9) |
| *Sertraline* | 22 (0.8) |
| *Duloxetine* | 19 (0.7) |
| *Escitalopram* | 15 (0.5) |
| *Venlafaxine* | 10 (0.3) |
| *Doxepin* | 9 (0.3) |
| *Desvenlafaxine* | 8 (0.3) |
| *Fluoxetine* | 7 (0.2) |
| *Paroxetine* | 3 (0.1) |
| *Clomipramin* | 3 (0.1) |
| *Nortriptyline* | 2 (0.1) |
| *Moclobemide* | 2 (0.1) |
| *Vortioxetine* | 1 (0.03) |
| *Mianserin* | 1 (0.03) |
| **Other psychotropic agents** |  |
| *Diazepam* | 105 (3.6) |
| *Temazepam* | 24 (0.8) |
| *Haloperidol* | 18 (0.6) |
| *Olanzapine* | 9 (0.3) |
| *Quetiapine* | 9 (0.3) |
| *Midazolam* | 9 (0.3) |
| *Oxazepam* | 8 (0.3) |
| *Clonazepam* | 8 (0.3) |
| *Alprazolam* | 6 (0.2) |
| *Nitrazepam* | 5 (0.2) |
| *Lorazepam* | 4 (0.1) |
| *Zolpidem* | 3 (0.1) |
| *Amisulpride* | 2 (0.1) |
| *Lithium* | 1 (0.03) |
| *Aripiprazole* | 1 (0.03) |
| *Clobazam* | 1 (0.03) |
| *Zopiclone* | 1 (0.03) |
